# Chasing Red Herrings: Palladium Metal Salt Impurities Feigning KRAS Activity in Biochemical Assays

**DOI:** 10.1021/acs.jmedchem.3c02381

**Published:** 2024-07-15

**Authors:** Thomas Gerstberger, Helmut Berger, Frank H. Büttner, Michael Gmachl, Dirk Kessler, Manfred Koegl, Simon Lucas, Laetitia J. Martin, Moriz Mayer, Darryl B. McConnell, Sophie Mitzner, Guido Scholz, Matthias Treu, Bernhard Wolkerstorfer, Stephan Zahn, Krzysztof M. Zak, Philipp A. Jaeger, Peter Ettmayer

**Affiliations:** ‡ Boehringer Ingelheim RCV GmbH & Co. KG, Dr. Boehringer Gasse 5-11, A-1121 Vienna, Austria; § Boehringer Ingelheim Pharma GmbH & Co. KG, Birkendorfer Str. 65, D-88397 Biberach, Germany

## Abstract

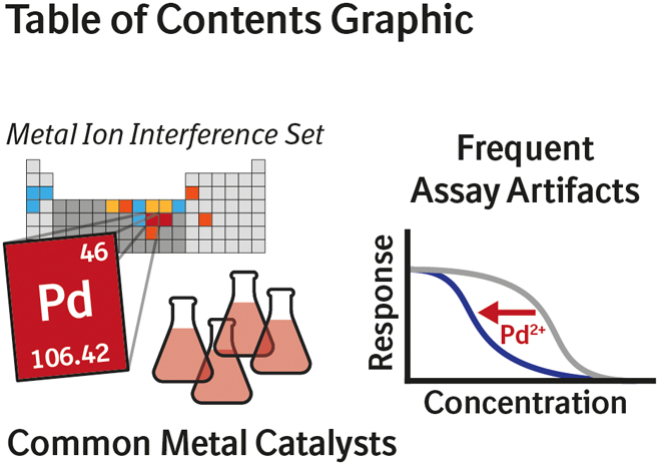

Identifying promising chemical starting points for small molecule inhibitors of active, GTP-loaded KRAS “on” remains of great importance to clinical oncology and represents a significant challenge in medicinal chemistry. Here, we describe broadly applicable learnings from a KRAS hit finding campaign: While we initially identified KRAS inhibitors in a biochemical high-throughput screen, we later discovered that compound potencies were all but assay artifacts linked to metal salts interfering with KRAS AlphaScreen assay technology. The source of the apparent biochemical KRAS inhibition was ultimately traced to unavoidable palladium impurities from chemical synthesis. This discovery led to the development of a Metal Ion Interference Set (MIIS) for up-front assay development and testing. Profiling of the MIIS across 74 assays revealed a reduced interference liability of label-free biophysical assays and, as a result, provided general estimates for luminescence- and fluorescence-based assay susceptibility to metal salt interference.

## Introduction

The Kirsten RAt Sarcoma viral oncogene homologue (KRAS) is associated with a high mortality rate, for example, in pancreatic ductal adenocarcinoma (PDAC), non-small cell lung cancer (NSCLC), and colorectal cancer (CRC).^1^ Functionally, KRAS cycles between an inactive, GDP-loaded (KRAS “off”) and an active, GTP-loaded (KRAS “on”) state, with the latter mediating downstream effector signaling.^2,3^

The majority of current clinical candidates target KRAS alleles in the GDP-loaded, KRAS “off” state and are thus vulnerable to shifts in the KRAS “on/off” equilibrium.^4−7^ As a consequence, the need for small molecule inhibitors that bind to the active GTP-loaded KRAS remains high. This study focuses on the unexpected finding of palladium (Pd) contamination feigning active hits in a KRAS AlphaScreen assay, leading to a broader discussion of interference of diverse metal salts with luminescent and fluorescent assays.

## Results and Discussion

### Unbiased, Biochemical High-Throughput Hit-Finding Campaign

Unbiased screening of large compound collections to identify chemical starting points remains a standard approach in small molecule drug discovery.^8,9^ Accordingly, we conducted a high-throughput screening (HTS) campaign on protein–protein interaction inhibitors for ten high-value targets, including KRAS “on”, in collaboration with FORMA Therapeutics. We screened approximately 1.7 million compounds in each target campaign, utilizing various combinations of cell-based MAPPIT,^10^ biochemical fluorescence polarization (FP),^11^ and AlphaScreen (AS)^12,13^ technologies for a total of over 15 million compound measurements. This collaboration resulted in highly validated chemical probes for the transcription factor BCL6^14^ and SOS1^15^ (BI-1136, BI-3802, BI-3812/BI-3406), all available freely at opnMe,^16^ as well as a clinical SOS1 inhibitor candidate.^17^

In the KRAS-targeting campaign, the full deck library yielded 6,917 confirmed KRAS “on” actives (0.4%; Z′ 0.86, full campaign triaging in Figure S1). We further evaluated these hits for dose-response in the screening (KRAS^G12D(GTP)^::CRAF AlphaScreen) and control assays to exclude singlet oxygen quenchers or nonspecifically interfering compounds (“Control assay” = AlphaScreen Shortcut). Out of 1,535 dose responsive and selective hits, we chose 87 cluster representatives and submitted them for validation by assessing KRAS or CRAF binding using saturation transfer difference NMR (STD-NMR) and thermal shift assay (TSA).

**Figure 1:**
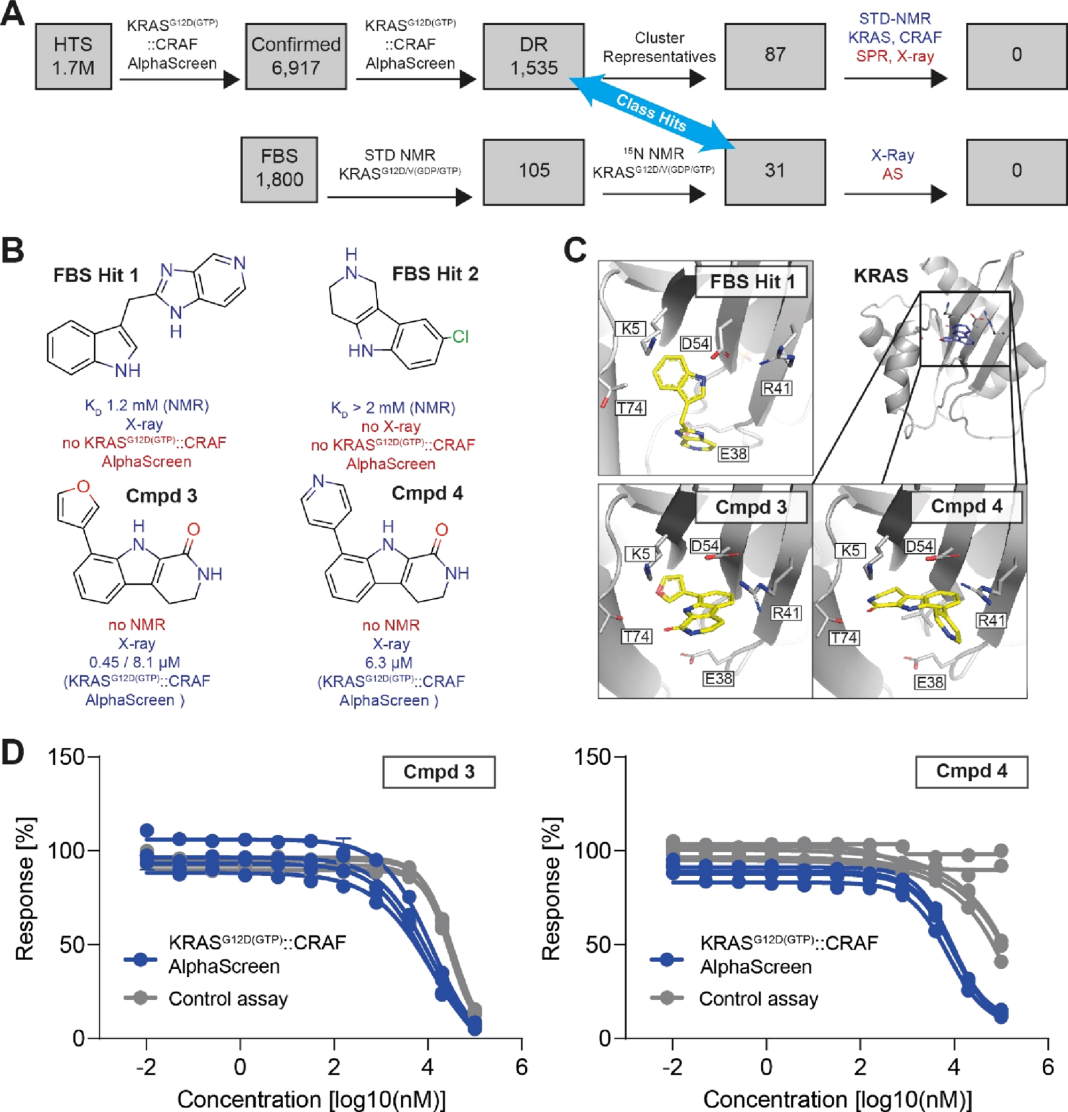
KRAS^G12D(GTP)^::CRAF hit finding. (A) Hit finding and triaging scheme for the High-Throughput Screen (HTS) and Fragment Based Screen (FBS). For details, see text. (B) Structures of the fragments found in the STD NMR screen (FBS Hits 1 and 2) as well as analogues that also showed effects in the HTS or subsequent AS screens (Cmpds 3 and 4). (C) X-ray structures of Cmpd 3 and Cmpd 4 bound to KRAS^G12D(GCP)^. (D) IC_50_ curves for KRAS^G12D(GTP)^::CRAF AlphaScreen (blue) and Control assay (gray). Data points indicate mean ± standard deviation of two technical replicates; curves indicate independent assay runs. For aggregate statistics, see Table 1.

Keenly aware of possible false-positive liabilities in large screening collections, we had checked all primary active hits against ”pan-assay interference compounds” (PAINS)^18,19^ and other known “compounds that interfere with assay technology” (CIAT)^20^ motifs. While we obtained seven singeltons with positive STD-NMR, none could be validated by SPR^5^ or X-ray crystallography, threatening to end this campaign without useful, validated hits (Figure 1A, top).

### Fragment-Based Screening Campaign Informs Hit Class Expansion from HTS

In parallel to the initial HTS approach, we had developed a nanomolar KRAS^G12D^ inhibitor (BI-2852, also available at opnMe) targeting the SI/II-pocket of KRAS by employing a structure-based drug design strategy originating from very weak-binding fragments.^21^ Building on this expertise, we again used NMR-based fragment screens (FBS), now against GDP- and nonhydrolyzable GCP-loaded KRAS^G12V/G12D^, which yielded NMR and X-ray validated hits with millimolar KRAS “on” affinities in NMR, but these hits now lacked activity in the biochemical KRAS^G12D(GTP)^::CRAF AlphaScreen (Figure 1A, bottom). Aiming to leverage hits and learnings from both the HTS and the FBS screen, we investigated structural similarities in the confirmed actives space and recognized shared “Class Hits” between both campaigns, complementing experimental results (Figure 1B). Binding of FBS Hit 1 in the KRAS SI/II-pocket was confirmed through X-ray crystallography; however, the binding mode of FBS Hit 2 remained elusive (Figure 1C, Table 1). We attributed this uncertainty to the weak binding affinity of FBS Hit 2 to KRAS. Employing the carboline structure of FBS Hit 2 in a substructure search led us to identify two additional carbolinone compounds (Cmpd 3 and Cmpd 4) with IC_50_ values of 0.4 μM (KRAS^G12D(GTP)^::CRAF AlphaScreen primary screen)/8.1 μM (confirmation screen) and 6.3 μM (confirmation screen only) in the KRAS^G12D(GTP)^::CRAF high-throughput AlphaScreen (Table 1). These compounds exhibited a small but robust potency window compared to the Control assay (Figure 1D). Motivated by the apparent enhancement of potency relative to the FBS Hits, we verified the interaction between KRAS and the ligand using STD-NMR (Table 1, Figures S2 and S3). Subsequently we came up with a reasonable binding mode of Cmpds 3 and 4 to the KRAS SI/II-pocket using protein crystallography, revealing that the two carbolinone analogs exhibited completely different binding modes and again different from FBS Hit 1. Cmpd 4 interacted via its carbolinone scaffold and formed a hydrogen bond with the pyridinone nitrogen to form Thr74. In contrast, Cmpd 3 formed an interaction with Thr74 via its furanyl oxygen. In both cases, the lipophilic carbolinone scaffold nestled against the upper region of the pocket (Figure 1C). Overall, these findings suggested a viable path forward toward expanding medicinal chemistry efforts on further hit expansion.

**Table 1:** Overview of the Hits from the Combined HTS and FBS Campaigns^a^

Cmpd	Batch	X-ray	KRAS^G12D(GTP)^::CRAF AlphaScreen IC_50_ [μM]	Control assay IC_50_ [μM]	Purification	KRAS STD-NMR	ICP-MS Pd [ppm]
FBS Hit 1	1	Yes	>100	>100	n.d.	n.d.	n.d.
FBS Hit 2	1	n.d.	>100	>100	n.d.	n.d.	n.d.
Cmpd 3	1	Yes	11.0 ± 1.5 (*n* = 4)	34.0 ± 2.6 (*n* = 4)	RP	11%	24,000
	2	n.d.	51.5 ± 3.5 (*n* = 6)	69.7 ± 6.7 (*n* = 6)	RP	n.d.	1,500
	3	n.d.	78.3 ± 2.9 (*n* = 2)	>100 (*n* = 2)	NP	n.d.	140
Cmpd 4	1	Yes	8.6 ± 0.8 (*n* = 4)	>100 (*n* = 5)	Filtration	7%	29,000
	2	n.d.	77.3 ± 55.7 (*n* = 6)	>100 (*n* = 8)	NP	n.d.	2,400
PdCl_2_	1	n.d.	0.7 ± 0.2 (*n* = 4)	5.8 ± 0.7 (*n* = 4)	n.d.	n.d.	n.d.

a All measurements are single measurements unless standard deviations are indicated; then average ± std were determined, as described in the Experimental Section.

### Analoging Revealed Erratic Structure–Activity Relationships and Resynthesis Led to Hit Devalidation

**Figure 2:**
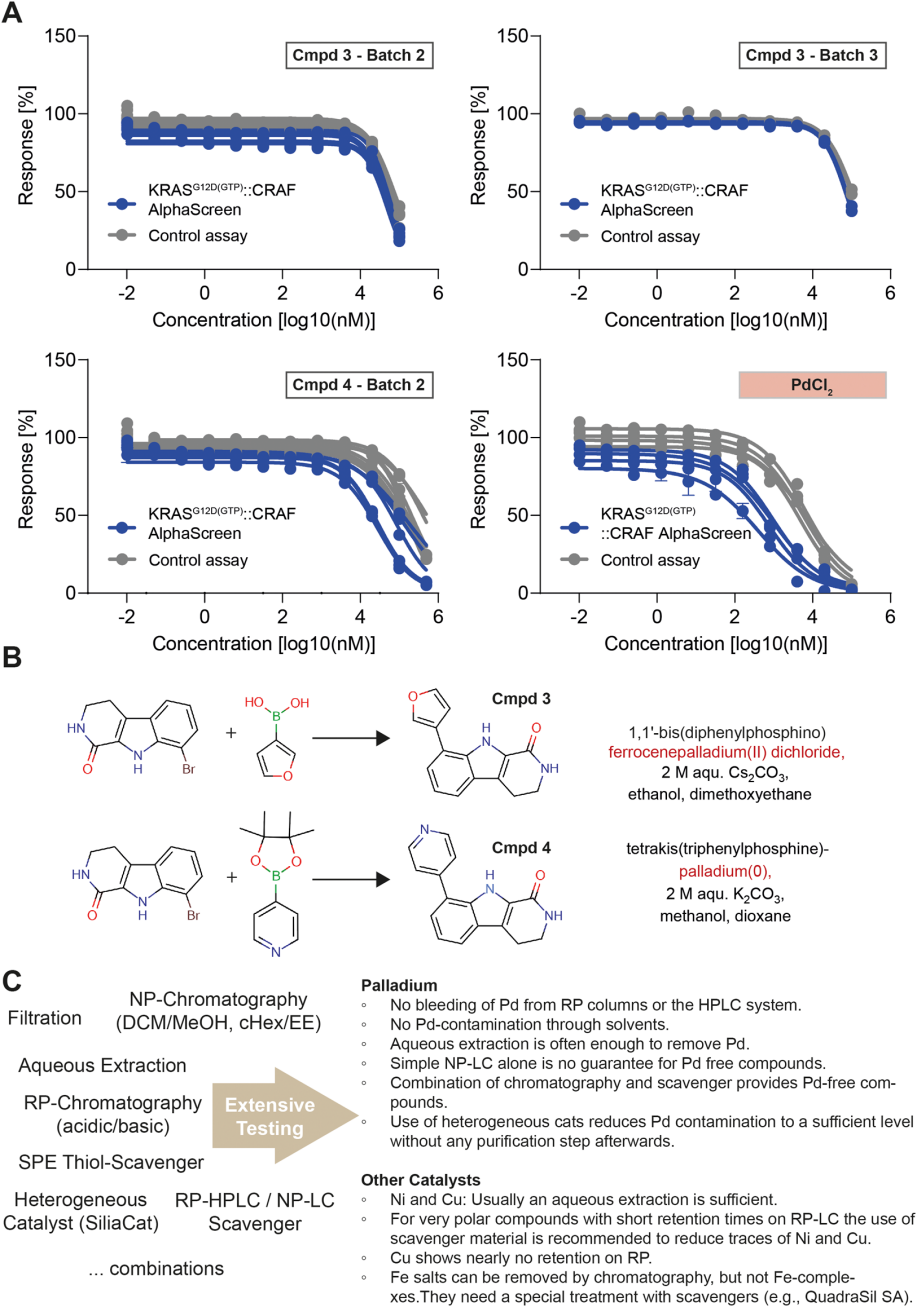
Effects of palladium contamination and possible solutions. (A) Resynthesized batches of Cmpd 3 and Cmpd 4 were purified differently than the original screening batch and showed a decreasingly smaller effect in the AlphaScreen assays. Ultimately, Pd was identified to drive the apparent inhibitory effect and affected both the KRAS^G12D(GTP)^::CRAF AlphaScreen and the Control assay. (B) Palladium could enter the compound samples at the cross-coupling catalysis step and was not removed completely during standard high-throughput chemistry purification steps. Possible synthesis routes shown for Cmpd 3 and 4. (C) As a workup to the failed lead identification, we conducted extensive testing of purification methods to arrive at generalizable guidelines for Pd-removal.

Despite a seemingly promising body of experimental evidence, structural similarities, and solved crystal structures, we were not able to replicate the inhibitory potency of Cmpd 3 and Cmpd 4 and close analogues in the KRAS^G12D(GTP)^::CRAF AlphaScreen, following compound resynthesis (Figure 2A, Table 1). Instead, a steep and unpredictable structure activity relationship raised concerns that unidentified confounding factors may have contributed to the apparent IC_50_ values. Due to prior experience in an internal Phosphoglycerate Dehydrogenase (PHGDH) project, where apparent enzymatic inhibition was traced to the presence of copper and mercury impurities,^22^ we measured the transition-state metal content of the first batches of the Cmpd 3 and Cmpd 4 and found 24,000 ppm and 29,000 ppm of Pd contamination respectively (Table 1). In line with the observed IC_50_ values for the contaminated first batches of Cmpd 3 and Cmpd 4, PdCl_2_ alone inhibited the KRAS^G12D(GTP)^::CRAF AlphaScreen and the Control assay with IC_50_ values of 0.7 and 5.8 μM respectively. Soberingly, all the observed potency of Cmpd 3 and Cmpd 4 in the biochemical assays could thus be explained by metal impurities resulting from the specifics of the high-throughput synthesis workflow. Although there is no clear evidence for metal binding sites on KRAS, metal cyclens have been described to bind to an allosteric site of RAS near the C-terminus.^23−25^ Binding to this pocket could explain the observed binding affinities by STD-NMR.^26^ The lack of potent binding of resynthesized/repurified Cmpd 3 and Cmpd 4 was later also confirmed by lack of shift perturbations at 1 mM in a ^15^N KRAS^G12D(GCP)^-bound HSQC NMR experiment (data not shown). The observation of crystals with bound ligands indicates that weak binding is possible in that pocket at mM concentrations, but structure driven hit expansion was not given priority for this project due to the ongoing efforts with BI-2852.^21^

### Necessity of Transition Metal Catalysis and Disadvantages in Downstream Screening

The formation of carbon–carbon bonds is an important aspect of modern organic synthesis frequently mediated by the employment of transition metal catalysts.^27^ In this specific case, Pd was used to catalyze the chemical synthesis of both compounds (Figure 2B). Eliminating transition metal contaminants from crude reaction products can pose significant challenges as complexes formed between metal impurities and the desired product may exhibit stable forms under various purification conditions (e.g., precipitation, crystallization, reversed phase, or normal phase chromatography). Metal impurities originating from commonly used synthetic organic chemistry catalysts are thus often unavoidable, even when utilizing state-of-the-art reversed phase chromatography techniques for purification, and can present significant removal challenges, also in industrial-scale pharmaceutical production workflows.^28^ Internally, we evaluated the deployment of metal scavenger cartridges and other metal removal approaches (data not shown) and identified solutions and practical guidelines (Figure 2C). However, those solutions are only possible to implement for repurification or resynthesis workflows and impractical to apply to million-compound libraries upfront. Consequently, a specific development of screening assays with reduced sensitivity to heavy metal impurities would be the most desirable solution, complemented by a development of robust metal-sensitivity-assessing test tools to evaluate current and future assay portfolios.

### Development and Testing of the Metal Ion Interference Set (MIIS)

Metal impurities leading to false positives in HTS campaigns have been described before.^29−31^ We intended to use our learnings to comprehensively identify the most robust assay formats with the goal of reducing the likelihood of false-positive results. We conducted a systematic comparison of different biochemical KRAS screening configurations (Table 2). We found that the minimalistic Control assay, consisting only of biotinylated GST, already exhibited substantial signal inhibition (IC_50_ 5.8 μM for PdCl_2_). This suggested a general sensitivity of luminescence based AlphaScreens toward Pd interference. This sensitivity was made significantly worse in the presence of tagged recombinant proteins in the KRAS^G12D(GTP)^::CRAF AlphaScreen assay (IC_50_ = 0.7 μM). Remarkably, switching the same tagged proteins to a fluorescence-based Time-Resolved Fluorescence Resonance Energy Transfer assay (TR-FRET assay) format led to a significant reduction in sensitivity to Pd by nearly 2 orders of magnitude (IC_50_ 40.5 μM), while preserving comparable positive control (recombinant CRAF binding) sensitivity observed in the original AlphaScreen (0.188 and 0.066 μM respectively). In this specific case, the TR-FRET assay thus demonstrates a clear advantage over Pd impurities, indicating that careful selection of robust assays across different settings should be possible for different screening formats. This assay- and protein configuration-specific liability led us to compile a collection of metal salts and incorporated this Metal Ion Interference Set (MIIS) into Boehringer Ingelheim′s assay interference set, which is routinely tested and evaluated prior to initiating extensive screening campaigns. The selection of metals was guided by their potential relevance in chemical synthesis of research compounds, their abundance and most relevant and stable oxidative state under physiological conditions, and the availability of respective salts with softness/polarizability, which could aid solubility in DMSO (Table 3). The latter led to Hg and Pt not being present in the MIIS. We identified DMSO soluble salts and prepared stock solutions for Pd^2+^, Au^3+^, Ag^1+^, Fe^2+^, Fe^3+^, Sn^2+^, Cu^2+^, Ni^2+^, Al^3+^, Mn^2+^, Zn^2+^, Co^2+^, Mg^2+^, and Ru^3+^. However, we subsequently observed instability in the Ru^3+^ DMSO solution and excluded it from the interference set.

**Table 2:** Palladium Interactions with the Assay Conditions^a^

Assay	Assay format	PdCl_2_ IC_50_ [μM]	CRAF(51–131) IC_50_ [μM]
KRAS^G12D(GTP)^::CRAF AlphaScreen^b^	Luminescence	0.7 ± 0.2 (*n* = 4)	0.2
Control assay^c^	Luminescence	5.8 ± 0.7 (*n* = 4)	n.d.
KRAS^G12D(GTP)^::CRAF TR-FRET^d^	Fluorescence	40.5 ± 19.2 (*n* = 4)	0.1

a Sensitivity of KRAS^G12D(GTP)^::CRAF assay formats and the Control assay to PdCl_2_ and untagged CRAF. Proteins used as follows:

b GST-CRAF^(1–303)^ and KRAS^G12D^(1–169)-biotin.

c Biotinylated GST.

d GST-CRAF^(51–131)^ and KRAS^G12D(1–169)^-biotin. All measurements are single measurements unless standard deviation is indicated, then average ± std were determined, as described in the Experimental Section.

**Table 3:** Metal Ion Interference Set (MIIS)^a^

Salt	Name	Exemplified relevant/frequent chemical reaction
PdCl_2_	Palladium(II) chloride	Cross-Coupling (e.g., Stille, Negishi, Heck), Reduction/Hydrogenation
AuCl_3_	Gold(III) chloride	Cross-Coupling, activation of C–C multiple bonds
Ag(CH_3_COO)	Silver acetate	Decarboxylation
FeCl_2_	Iron(II) chloride	Reduction/Hydrogenation
FeCl_3_	Iron(III) chloride	Reduction/Hydrogenation
SnCl_2_	Tin(II) chloride	Reduction, Stille-Cross-Coupling
CuCl_2_	Copper(II) chloride	1,4-Additions, Sonogashira Cross-Coupling, Huisgen-Reaction (Click-Chemistry)
NiCl_2_	Nickel(II) chloride	Reduction/Hydrogenation, Kumada-Coupling
Al(NO_3_)_3_	Aluminum nitrate	Friedel–Crafts reaction
MnCl_2_	Manganese(II) chloride	Oxidation
ZnCl_2_	Zinc(II) chloride	Negishi-Coupling
CoCl_2_	Cobalt(II) chloride	Raney-Cobalt, hydration of nitriles
MgCl_2_	Magnesium chloride	Kumada-Coupling
KCl	Potassium chloride	Commonly used assay buffer ingredients
NaCl	Sodium chloride	Commonly used assay buffer ingredients
NH_4_Cl	Ammonium chloride	Commonly used assay buffer ingredients
RuCl_3_^b^	Ruthenium(III) chloride	Reduction/Hydrogenation, Olefin-Metathesis

a Each salt as a 10 mM stock solution, as described in the Experimental Section.

b Later excluded due to instability of the DMSO solution.

We have now collected data for MIIS from 74 biochemical and biophysical assays encompassing a diverse array of assay technologies and targets (Table 4).

**Table 4:** Metal Ion Interference Set (MIIS) Test and Target Set^a^

Master assay	Assay technology	Target and/or assay published by Boehringer Ingelheim	# of targets
Luminescence	AlphaScreen	Undisclosed	2
Luminescence	Luminescence	Undisclosed	1
Luminescence	AlphaScreen	Undisclosed	2
Luminescence	Luminescence	Undisclosed	3
Luminescence	AlphaScreen	Undisclosed	1
Luminescence	ADP-Glo	Undisclosed	1
Luminescence	AlphaScreen	FKBP51^32^	3
Luminescence	AlphaScreen	Undisclosed	1
Luminescence	Luminescence	Undisclosed	1
Luminescence	AlphaScreen	KRAS^21^	3
Luminescence	Luminescence	Undisclosed	1
Luminescence	AlphaScreen	MDM2^33^	3
Luminescence	AlphaScreen	Undisclosed	2
Luminescence	ADP-Glo	Undisclosed	10
Luminescence	AlphaScreen	Undisclosed	4
Luminescence	AlphaScreen	Undisclosed	2
Luminescence	AlphaScreen	SOS1^34^	2
Luminescence	AlphaScreen	Undisclosed	1
Luminescence	AlphaScreen	UPA-UPAR^35^	2
Luminescence	ADP-Glo	WRN^36^	4
Fluorescence	Fluorescence	Undisclosed	1
Fluorescence	DELFIA	Undisclosed	1
Fluorescence	TR-FRET	Undisclosed	2
Fluorescence	Fluorescence Polarization	BCL6^37^	1
Fluorescence	TR-FRET	beta-Catenin^38^	1
Fluorescence	Fluorescence	Undisclosed	1
Fluorescence	TR-FRET	SOS1^34^	1
Fluorescence	TR-FRET	Undisclosed	1
Fluorescence	TR-FRET	KRAS^21^	1
Fluorescence	TR-FRET	Undisclosed	1
Fluorescence	DELFIA	LSD1^39^	1
Fluorescence	TR-FRET	NSD3^40^	1
Fluorescence	TR-FRET	Undisclosed	1
Fluorescence	TR-FRET	Undisclosed	1
Biophysical	MALDI	Undisclosed	1
Biophysical	MALDI	Undisclosed	2
Biophysical	MALDI	Undisclosed	1
Biophysical	RapidFire MS	Undisclosed	1
Biophysical	MALDI	Undisclosed	1
Biophysical	MALDI	EGFR^41^	1
Biophysical	MALDI	Undisclosed	1
Biophysical	MALDI	Undisclosed	2

a Master assay technologies used in this study and (disclosed) targets. Number of assays for the listed target project include screen and counter screens.

### A General Advantage of Reduced Interference-Liability of Label-Free Biophysical Assays

**Figure 3:**
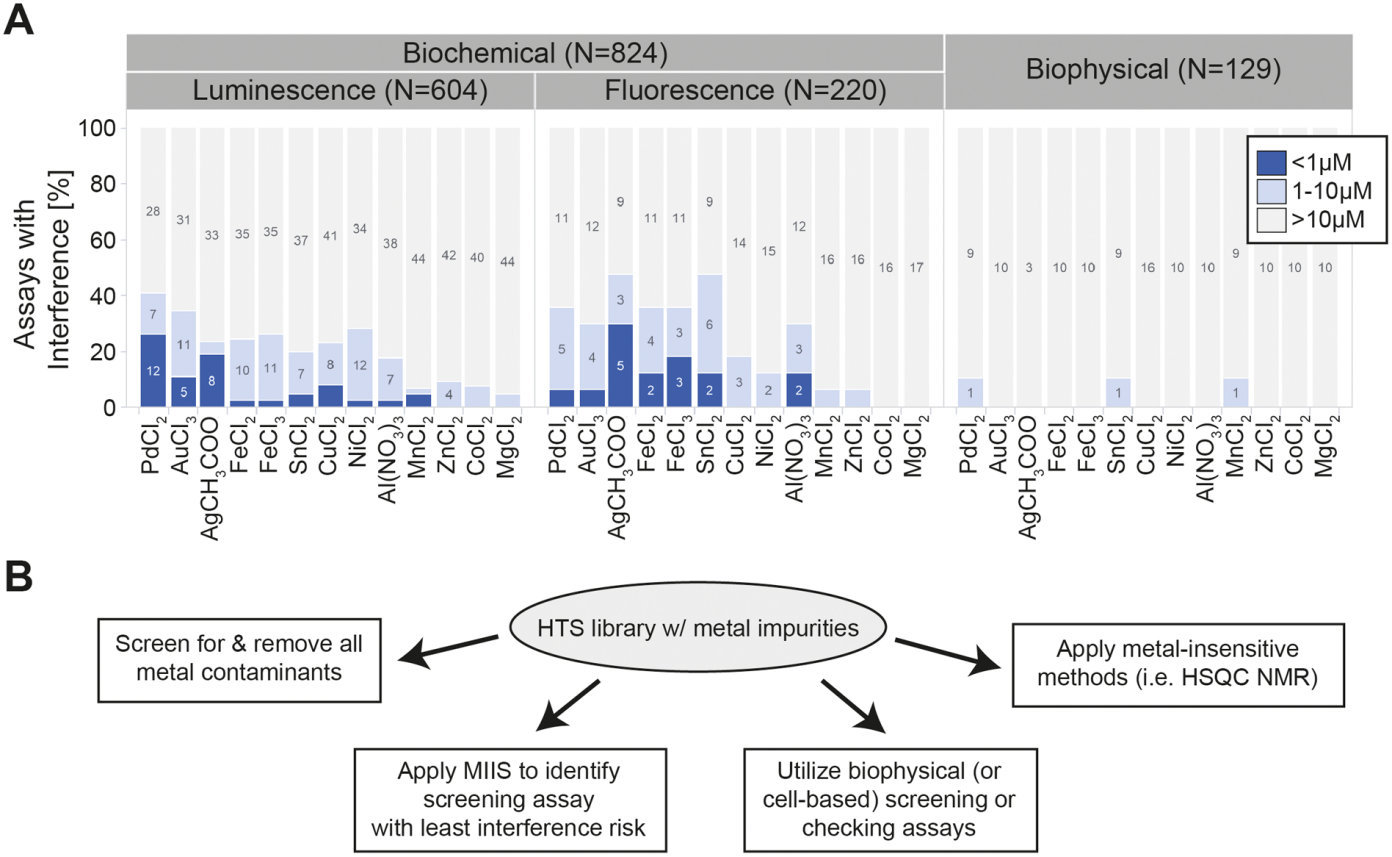
Performance characteristics of MIIS across a large assay set (see Table 4). (A) Sensitivity of tested master assays against the MIIS set, bound by observed IC_50_. Numbers indicate assay/target combinations tested. (B) Overview of the possible approaches to minimize transition metal interference in hit finding campaigns.

While we detected high inhibition (IC_50_ < 1 μM) for some transition metals such as Pd^2+^, Au^3+^ and Ag^1+^ in approximately 16% of all tested assay/target combinations, other metals–for example Mn^2+^, Zn^2+^, Co^2+^ and Mg^2+^ – had low apparent impact on assay read-out (Figure 3A). Remarkably, however, every single metal salt tested caused interference in at least two assays with moderate potency (IC_50_ < 10 μM), highlighting the prevalence of this issue, especially in low potency hit-finding campaigns. Since Pd catalysts are most frequently applied in high-throughput cross-coupling reactions for large compound collections, we believe that this is the metal to pay the closest attention to. In line with our KRAS^G12D(GTP)^::CRAF example, metal ion interference was not linked to the assay technology alone: both luminescence- and fluorescence-based assays are equally affected by metal ions (Figure 3A). Interestingly the biophysical assays (i.e., MALDI or RapidFire MS) are much less prone to metal ion interference - a reassuring observation highlighting the advantages of label free screening technologies in modern drug discovery.

While cellular assays are less common in initial hit finding screens and thus less frequently exposed to potentially contaminated HTS library compounds, we also tested the MIIS in various cell culture setups for effects on both viability and reporter systems. We did not observe any effects on cytotoxicity measured by CellTiter-Glo across five common cancer cell lines (NCI-H358, SW620, HEK293, NCI-H2052, JURKAT E6), nor for the popular luminescent Nano-Glo HiBit signal for different reporter proteins in these same cell lines (nM to μM concentration range, data not shown). More generally, whenever we test the MIIS in cellular assays, with both fluorescent and luminescent readouts, we observe a much lower interference rate than for biochemical, but not as low as in biophysical assays: only 28 of 444 cellular assays (6.3%, data not shown) showed MIIS IC_50_ inhibition less than 10 μM, while 173 of 824 (20.9%) did so in biochemical and 3 of 129 (2.3%) for biophysical assays (Figure 3A).

## Conclusions

The development of small molecule inhibitors for active GTP loaded KRAS “on” remains an important but challenging task, requiring combinations of various approaches, such as NMR-based fragment screens, structure-based design methods, and high-throughput hit finding campaigns. However, the presence of metal impurities such as palladium (Pd) resulting from chemical synthesis of the compounds in high-throughput screening decks can lead to false positive hits, as transition metals can interfere in often unforeseeable ways with assay principles and assay reagents. Having learned from a sobering negative hit finding experience and a comprehensive failure workup, we here describe the development of a simple, yet versatile metal ion interference set (MIIS). We propose to use this set extensively during assay development to experimentally test for confounding factors and adjust the hit finding strategies accordingly. Furthermore, standardization of an early follow-up with biophysical methods can further reduce the interference risk of metal impurities, thus significantly improving the likelihood of successfully completing screening campaigns and avoiding costly and unsuccessful detours. Overall, we can now point at an available selection of alternative or combined approaches to minimize interference risks (Figure 3B). Although our KRAS-CRAF example did not lead to the discovery of any new useful chemical matter in the KRAS Switch I/II pocket, it provided valuable insights on how to optimize assay validation and assays for future screening efforts and has already contributed positively to many internal campaigns. By reducing the potential impact of metal impurities and enhancing the accuracy of screening campaigns overall, we aim to improve the efficiency and success rate of future drug discovery efforts, which are still major cost-drivers across the pharmaceutical industry. We believe that these advances will contribute to the future development of novel therapeutics targeting challenging proteins, such as, but not limited to, active GTP loaded KRAS “on”.

## Experimental Section

### General Section

If not explicitly mentioned otherwise, all samples showed a purity of ≥95%. Samples were analyzed on an Agilent 1200 series LC system coupled to an Agilent 6140 mass spectrometer. Purity was determined via UV detection with a bandwidth of 170 nm in the range from 230 to 400 nm. LC parameters were as follows: Waters Xbridge C18 column, 2.5 μm particle size, 2.1 × 20 mm2 or 3.5 μm particle size, 2.1 × 30 mm^2^, run time 2.1 min, flow 1 mL/min, column temperature 60 °C, and 5 μL injections. Solvent A (20 mM NH_4_HCO_3_/NH_3_; pH 9), solvent B (MS grade acetonitrile). Start 10%B, gradient 10–95% B from 0.0 to 1.5 min, 95% B from 1.5 to 2.0 min, gradient 95–10% B from 2.0 to 2.1 min.

### Statistics

For aggregated summary statistics, multiple measurements were aggregated to mean and standard deviation, respectively, using the following formulas:Mean:
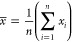
Standard Deviation:
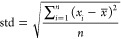


### Synthetic Procedures

#### 8-(Furan-3-yl)-2H,3H,4H,9H-pyrido[3,4-*b*]indol-1-one (**3**, batch 1)

8-Bromo-2,3,4,9-tetrahydro-1H-pyrido[3,4-*b*]indol-1-one (80 mg, 0.30 mmol), furane-3-boronic acid (40 mg, 0.36 mmol), and tetrakis(triphenylphosphine)palladium (0) (2 mg, 2 μmol) in methanol (300 μL)/dioxane (300 μL)/2 M aqueous potassium carbonate solution (300 μL) were stirred in a microwave reactor for 20 min at 120 °C. Water (100 μL) and trifluoroacetic acid (100 μL) were added, and the mixture was purified by prep. RP-HPLC/MS to give 23 mg **3** (30%).

#### 8-(Furan-3-yl)-2H,3H,4H,9H-pyrido[3,4-*b*]indol-1-one (**3**, batch 2)

8-Bromo-2,3,4,9-tetrahydro-1H-pyrido[3,4-*b*]indol-1-one (100 mg, 0.38 mmol), furane-3-boronic acid (51 mg, 0.45 mmol), cesium carbonate (368 mg, 1.31 mmol), and 1,1′-bis(diphenylphosphino)ferrocenepalladium(II) dichloride and dichloromethane (32 mg, 38 μmol) in N,N-dimethylformamide (1 mL)/water (1 mL) were stirred for 1 h at 90 °C. The reaction mixture was filtered and purified by prep. RP-HPLC/MS to yield 25 mg **3** (26%).

#### 8-(Furan-3-yl)-2H,3H,4H,9H-pyrido[3,4-*b*]indol-1-one (**3**, batch 3)

The product obtained using the procedure above was dissolved in dichloromethane and purified by column chromatography: 4 g SiO_2_, solvent A: dichloromethane, solvent B: methanol, gradient: 0–1 min 0% B, 1–12 min 0–5% B, 12–13 min 5% B. Yield: 25 mg (quant.)

^1^H NMR (DMSO-*d*_6_, 500 MHz) δ 8.43 (s, 1H), 7.79 (t, 1H, J = 1.6 Hz), 7.62 (br s, 1H), 7.57 (d, 1H, J = 7.9 Hz), 7.42 (dd, 1H, J = 0.9, 7.3 Hz), 7.15 (t, 1H, J = 7.7 Hz), 7.01 (dd, 1H, J = 0.8, 1.7 Hz), 3.52 (dt, 2H, J = 2.7, 6.9 Hz), 2.94 (t, 2H, J = 6.9 Hz).

^13^C NMR (DMSO-*d*_6_, 126 MHz) δ: 161.4, 143.5, 140.6, 133.9, 128.1, 126.2, 123.4, 122.1, 120.1, 119.7, 119.2, 117.5, 110.0, 40.9, 20.3.

#### 8-(Pyridin-4-yl)-2H,3H,4H,9H-pyrido[3,4-*b*]indol-1-one (**4**, batch 1)

8-Bromo-2,3,4,9-tetrahydro-1H-pyrido[3,4-*b*]indol-1-one (80 mg, 0.30 mmol), 4-(4,4,5,5-tetrameythyl-1,3,2-dioxaborolan-2-yl)pyridine (73 mg, 0.36 mmol), and tetrakis(triphenylphosphine)palladium (0) (2 mg, 2 μmol) in methanol (300 μL)/dioxane (300 μL)/2 M aqueous potassium carbonate solution (300 μL) were stirred in a microwave reactor for 20 min at 120 °C. Water (100 μL) and trifluoroacetic acid (100 μL) were added. The precipitate was collected by filtration, triturated with water and dried in vacuo to yield 48 mg **4** (61%).

#### 8-(Pyridin-4-yl)-2H,3H,4H,9H-pyrido[3,4-*b*]indol-1-one (**4**, batch 2)

8-Bromo-2,3,4,9-tetrahydro-1H-pyrido[3,4-*b*]indol-1-one (1.00 g, 3.77 mmol), pyridine-4-boronic acid (556 mg, 4.53 mmol), and 1,1′-bis(diphenylphosphino) ferrocenepalladium(II) dichloride in 2 M aqueous cesium carbonate solution (3.77 mL, 7.54 mmol)/dimethoxyethane (5 mL) were stirred for 2 h at 80 °C. The reaction mixture was partitioned between water and dichloromethane, and the aqueous layer was extracted exhaustively. The combined organic layer was concentrated and purified by column chromatography: 40 g SiO_2_, solvent A: dichloromethane, solvent B: methanol, gradient: 0–1 min 0% B, 1–12 min 0–5% B, 12–13 min 5% B. Yield: 542 mg (55%).

^1^H NMR (DMSO-*d*_6_, 500 MHz) δ 11.43 (s, 1H), 8.65 (d, 2H, J = 5.7 Hz), 7.70 (d, 1H, J = 7.9 Hz), 7.6–7.6 (m, 3H), 7.32 (d, 1H, J = 6.9 Hz), 7.2–7.3 (m, 1H), 3.52 (dt, 2H, J = 2.4, 6.9 Hz), 2.96 (t, 2H, J = 6.8 Hz).

^13^C NMR (DMSO-d_6_, 126 MHz) δ: 161.8, 150.3, 145.9, 134.8, 129.0, 126.8, 125.1, 124.5, 124.0, 121.4, 120.6, 119.8, 41.4, 20.8.

#### MIIS Stock Solutions

Metal salt solutions for the metal ion interference set (MIIS) were prepared as 10 mM stock solutions in DMSO (see Table 3). All metal salts used have been purchased at highest industrial grade.

#### KRAS^G12D(GTP)^::CRAF AlphaScreen

This assay measures the inhibitory effect of compounds on KRAS^G12D^ and CRAF protein–protein interactions in the presence of GTP using AlphaScreen technology by PerkinElmer. The proteins have been used at these given final assay concentrations:

KRAS^G12D^ 1–169, N-terminal 6His-tag, C-terminal avi-tag (Xtal BioStructures, Inc.); final assay concentration 30 nM; CRAF 1–303, GST-tag, TEV cleavage site (Viva Biotech Ltd.); final assay concentration 4 nM.

Test compounds dissolved in DMSO were dispensed onto assay plates (Proxiplate 384 PLUS, white, PerkinElmer; 6008289) using an Access Labcyte Workstation with the Labcyte Echo 555. For the chosen highest assay concentration of 100 μM, 150 nl of compound solution were transferred from a 10 mM DMSO compound stock solution. A series of 11 5-fold dilutions per compound was transferred to the assay plate, and compound dilutions were tested in duplicates. DMSO was added as backfill to a total volume of 150 nl. The assay was run on a fully automated robotic system in a darkened room below 100 Lux. To 150 nl of compound dilution 10 μL of a mix including KRAS^G12D^, CRAF (final assay concentrations see above) and GTP nucleotide (Sigma G8877; final assay concentration 300 nM) in assay buffer (1 x PBS, 0.1% BSA, 0.05% Tween 20) were added into columns 1–24. After 60 min incubation time 5 μL of AlphaScreen bead mix in assay buffer were added into columns 1–23. Bead mix consisted of AlphaLISA Glutathione Acceptor Beads (PerkinElmer, cat. no. AL109) and Alpha-Screen Streptavidin Donor Beads (perkinElmer cat. no. 6760002) in assay buffer at a final assay concentration of 10 μg/mL each. Plates were kept at room temperature in a darkened incubator. After an additional 60 min incubation time the signal was measured on a PerkinElmer Envision HTS Multilabel Reader using the AlphaScreen specs from PerkinElmer.

Each plate contained 16 wells of a negative control (diluted DMSO instead of test compound; with KRAS^G12D(GTP)^::CRAF mix and bead mix; column 23) and 16 wells of a positive control (diluted DMSO instead of test compound; with KRAS^G12D(GTP)^::CRAF mix without bead mix; column 24). IC_50_ values were calculated and analyzed with Boehringer Ingelheim’s MEGALAB IC_50_ application using a 4 parametric logistic model.

#### TR-FRET Assay Technology

The proteins were used at these final assay concentrations:

KRAS^G12D^ 1–169, N-terminal 6His-tag, C-terminal avi-tag (Xtal BioStructures, Inc.); final assay concentration 15 nM, CRAF Ras binding domain, GST-tag, TEV cleavage site (Boehringer Ingelheim in house protein preparation); final assay concentration 15 nM.

Test compounds were dissolved in DMSO as described above. The assay was run on a fully automated robotic system. To 150 nl of compound dilution 15 μL of a mix including KRAS^G12D^, CRAF (final assay concentrations see above), GTP nucleotide (Sigma G8877; final assay concentration 10 μM), Lance Eu–W1024 labeled Streptavidin (PerkinElmer, Cat No AD0062; final assay concentration 1.5 nM) and Anti-GST surelight APC (PerkinElmer, Cat No AD0059G; final assay concentration 30 nM) in assay buffer (1 x PBS, 0.1% BSA, 0.05% Tween 20) were added into columns 1–23 and 15 μL of the solution without KRAS^G12D^ were added to row 24. Plates were kept at room temperature in a darkened incubator. After 60 min incubation time, the signal was measured on a PerkinElmer Envision HTS Multilabel Reader using the TR-FRET LANCE Ultra specs from PerkinElmer.

Each plate contained 16 wells of a negative control (diluted DMSO instead of test compound; with KRAS^G12D^; column 23) and 16 wells of a positive control (diluted DMSO instead of test compound; without KRAS^G12D^; column 24).

IC_50_ values were calculated and analyzed with Boehringer Ingelheim’s MEGALAB IC_50_ application using a 4 parametric logistic model.

#### AlphaScreen Shortcut [“Control Assay″]

This assay was developed to identify compounds that interfere with the assay format using AlphaLISA Glutathione Acceptor and AlphaScreen Streptavidin Donor beads. The assay was performed as described above using biotinylated GST as bridging reagent instead of KRAS^G12D^ and CRAF mix.

#### STD NMR

Experiments were recorded with a saturation time Tsat of 2 s and a total recycle delay of 3.6 s with the standard Bruker pulse program stddiffgp19.2. The shaped pulse train was made of G4 pulses with a length of 50 ms at a power of 31 μW. Here on resonance saturation was applied at 100 Hz and off resonance at 20000 Hz. No spin locking was applied to reduce the protein background. Here, 256 scans were applied with a time domain of 32 000 and the watergate delay was set to 90 μs to reduce remaining HDO signal intensity. 10 μM KRAS^G12D(GCP)^ and 1000 μM ligand was added for the STD NMR experiments and the percent enhancements approximated visually since no additional analysis was performed.

#### ICP MS

We set up a method to quantify metal impurities that were included in the DMSO stock solutions of the respective compounds tested. No effort was made to bring possible insoluble metal components into solution, as we were mainly interested in the soluble metal impurities and salts that potentially interfere with biophysical or biochemical assays. The intended final compound concentration was 300 μM which was obtained as follows: 30 μL of the standard 10 mM DMSO stock solution were diluted with 170 μL of pure DMSO, 50 μL of Internal Standard solution (in most cases indium), and 750 μL of a freshly prepared 2% HNO_3_ solution yielding a total of 1000 μL that was used for analysis. The final solution was filtered through a 0.45 μm cellulose filter prior to analysis to prevent clogging of the autosampler or nebulizer.

An Agilent 7900 ICP-MS with an integrated autosampler with the settings as noted in Table 5 was used:

**Table 5:** 

Parameter	Value	Units
RF power	1550	W
RF matching	1.80	V
Sample depth	10	Mm
Carrier gas	0.88	L/min
Makeup gas	0.10	L/min
Nebulizer pump	0.10	Rps
S/C temp	5	°C
Sample analysis time	1	min
Nebulizer	Miramist	
Cone	Pt-sampling cone	

Data was evaluated with Agilent’s Mass Hunter software (V.4.1), and quantitation of Pd was obtained from calibration curves determined on the same day with Indium or Gallium as internal control standards. The threshold of quantitation for Pd depended on how well it was detected by ICP MS and its natural occurrence as a ubiquitous contaminant in our setup. This value was represented in background equivalent concentrations (BEC) and was determined for our specific setup. Pd contamination was reported in mol % relative to the compound under the assumption that the compound stock solutions were 10 mM. If the Pd impurity exceed the BEC of 0.01 mol %, they are reported as is, otherwise they are reported as <0.01 mol %.

#### Protein Crystallization and Structure Determination

Apo protein crystals were grown in solution consisting of 53.4% (v/v) 2-methyl-2,4-pentanediol and 0.05 M MES pH 6.4 at 4 °C and then transferred into soaking buffer containing compound solution supplemented with 66% MPD, 53.4% (v/v) 2-methyl-2,4-pentanediol and 0.05 M MES pH 6.5. Crystals were soaked for 1 and 3 days for compounds Cmpd 4 and Cmpd 3, respectively, followed by flash freezing in liquid nitrogen. Data were collected at the SLS beamline X06SA (Swiss Light Source, Paul Scherrer Institute; wavelength of 1 Å using the PILATUS 6M-F detector) and images were processed with autoPROC.^42^ The resolution limits were set using default autoPROC settings using STARANISO for anisotropic resolution cutoff. The structures were solved by molecular replacement using the KRAS structure 4EPV as a search model. Subsequent manual model building, and refinement was done using COOT^43^ and autoBUSTER.^44^ Final models were deposited in the PDB as entries 8R7W for Cmpd 3 and 8R7X for Cmpd 4.

## Supporting Information Available

Full triaging scheme of the HTS campaign, spectra and detailed disclosure of MIIS IC_50_ values across targets published by Boehringer Ingelheim (PDF)

## Abbreviations

AS: AlphaScreen
CIAT: compounds that interfere with assay technology
CRC: colorectal cancer
DMSO: dimethyl sulfoxide
FBS: fragment based screening
FP: fluorescence polarization
GDP: guanosine diphosphate
GTP: guanosine triphosphate
HSQC: heteronuclear single quantum coherence
HTS: High throughput screening
IC_50_: 50% inhibitory concentration
ICP-MS: Inductively Coupled Plasma-Mass Spectrometry
KRAS: Kirsten rat sarcoma
NSCLC: nonsmall cell lung cancer
MAPPIT: mammalian protein–protein interaction trap
MIIS: metal ion interference set
NMR: nuclear magnetic resonance
MS: mass spectrometry
NP: normal-phase chromatography
n.d.: not determined
PAINS: pan-assay interference compounds
PDAC: pancreatic ductal adenocarcinoma
RP: reversed-phase liquid chromatography
SOS1: son-of-sevenless homologue 1
SPR: surface plasmon resonance
STD-NMR: saturation transfer difference nuclear magnetic resonance
TSA: thermal shift assay;
