# Supplementary material for: Chasing Red Herrings: Palladium Metal Salt Impurities Feigning KRAS Activity in Biochemical Assays
Source: J Med Chem. 2024 Jul 15;67(14):11701–11. doi: 10.1021/acs.jmedchem.3c02381 (PMC11285368; doi:10.1021/acs.jmedchem.3c02381)
Supplement: Supplementary file 1 — jm3c02381_si_001.pdf [file jm3c02381_si_001.pdf]

# Supporting Information

# Chasing Red Herrings: Palladium Metal Salt Impurities Feigning KRAS Activity in Biochemical Assays

Thomas Gerstberger[*‡], Helmut Berger[‡], Frank H. Büttner[§], Michael Gmachl[‡], Dirk Kessler[‡], Manfred Koegl[‡], Simon Lucas[‡#], Laetitia J. Martin[‡±], Moriz Mayer[‡], Darryl B. McConnell[‡+], Sophie Mitzner[‡], Guido Scholz[‡], Matthias Treu[‡], Bernhard Wolkerstorfer[‡], Stephan Zahn[‡], Krzysztof M. Zak[‡], Philipp A. Jaeger[*‡], Peter Ettmayer[*‡]

[‡]Boehringer Ingelheim RCV GmbH & Co. KG, Dr. Boehringer Gasse 5-11, A-1121 Vienna, Austria
[§]Boehringer Ingelheim Pharma GmbH & Co. KG, Birkendorfer Str. 65, D-88397 Biberach, Germany
[#]Present Address: Merck KGaA, Frankfurter Str. 250, D-64293 Darmstadt, Germany
[±]Present Address: F. Hoffmann-La Roche Ltd, Grenzacherstrasse 124, CH-4070 Basel, Switzerland
[+]Present Address: Curie Bio LLC, 196 Broadway, 1st Floor, Cambridge MA 02139, USA

[*]Corresponding authors: thomas.gerstberger@boehringer-ingelheim.com; philipp.jaeger@boehringer-ingelheim.com; peter.ettmayer@boehringer-ingelheim.com;

**Table of Contents:**

FIGURE S1. Full triaging scheme of the HTS campaign S2
FIGURE S2. $^{1}$H NMR and STD NMR spectra of Cmpd **4** S2
FIGURE S3. $^{15}$N HSQC spectra S3
FIGURE S4. $^{1}$H and $^{13}$C NMR spectra S4
FIGURE S5. HPLC chromatograms S5
Supplementary Table S1. Disclosed target list with $IC_{50}$ values for measured MIIS S6
Supplementary Table S2. Xray table S9
References S10

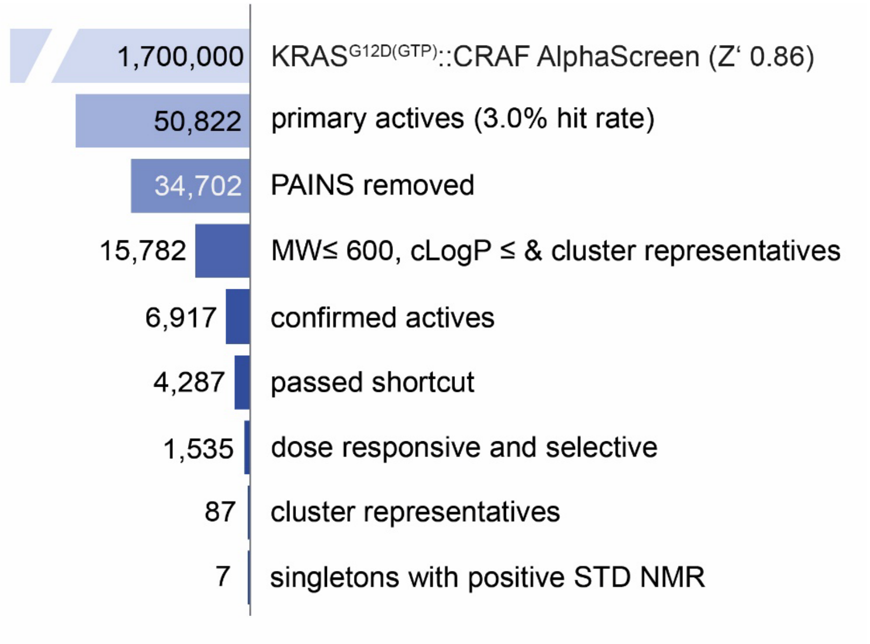


FIGURE S1. Full triaging scheme of the HTS campaign.

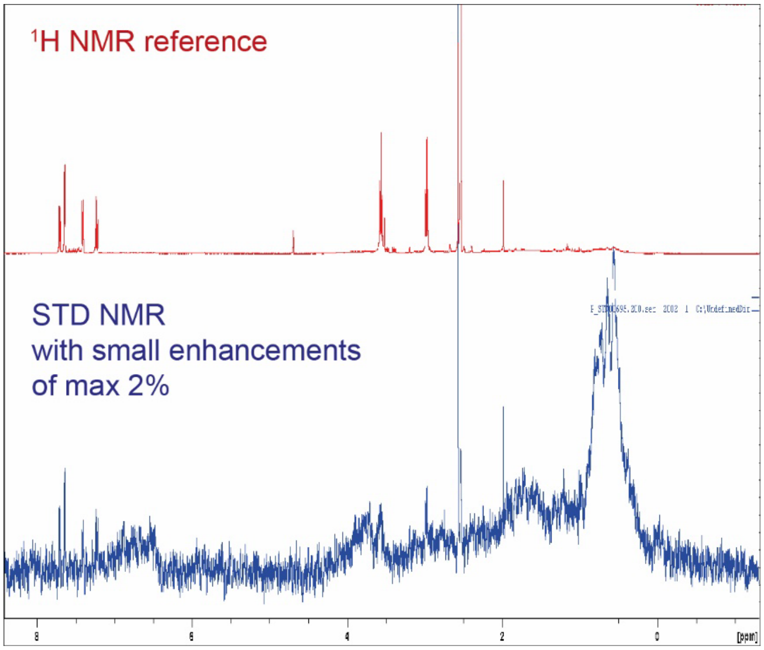


FIGURE S2. $^{1}$H NMR and STD NMR spectra of Cmpd **4** showing small enhancements.

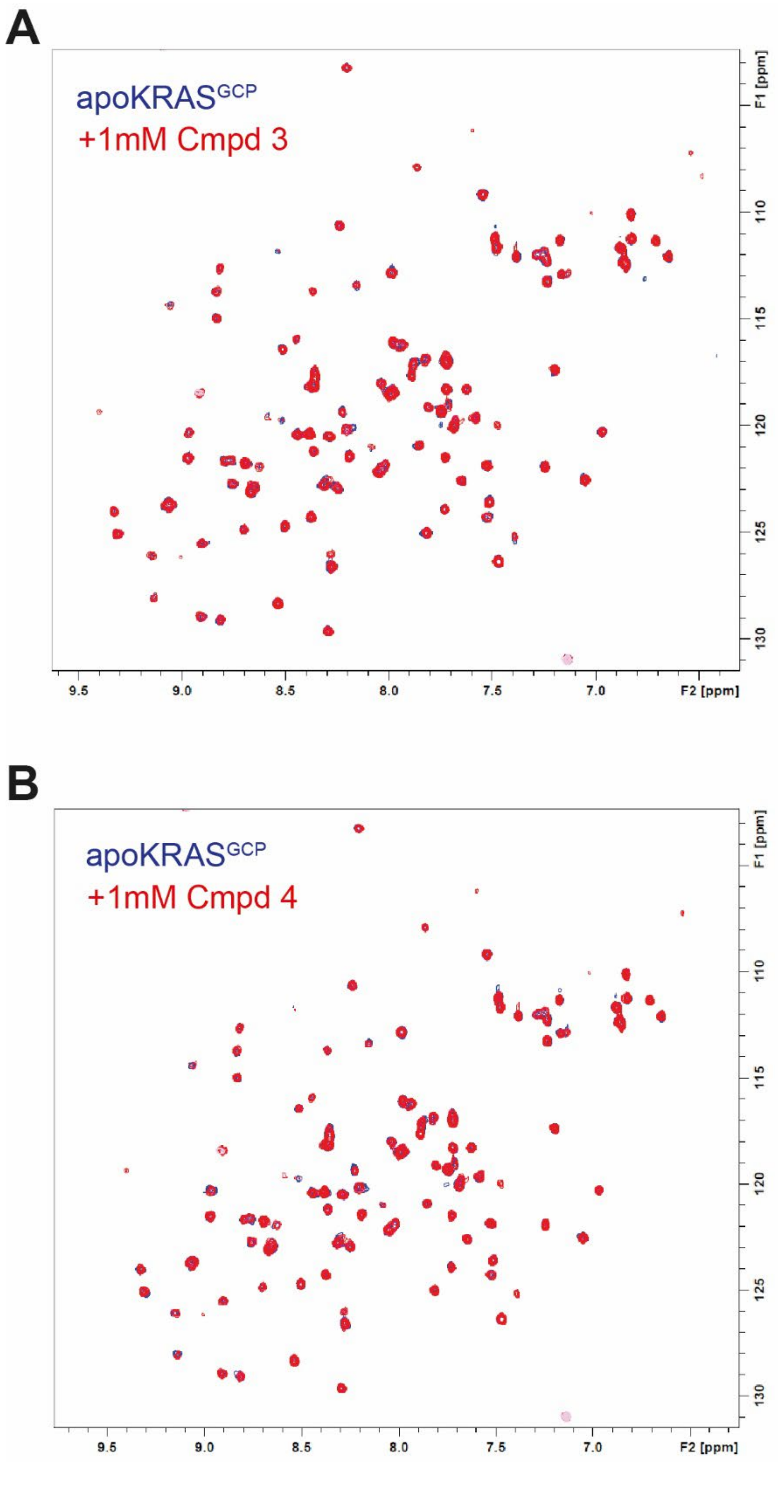


FIGURE S3. $^{15}$N HSQC spectra of 70µM KRAS in presence of (A) 1 mM Cmpd **3** and (B) 1 mM Cmpd **4** showing that no chemical shift perturbations (CSPs) were induced at these concentrations, indicating they are extremely weak binders.

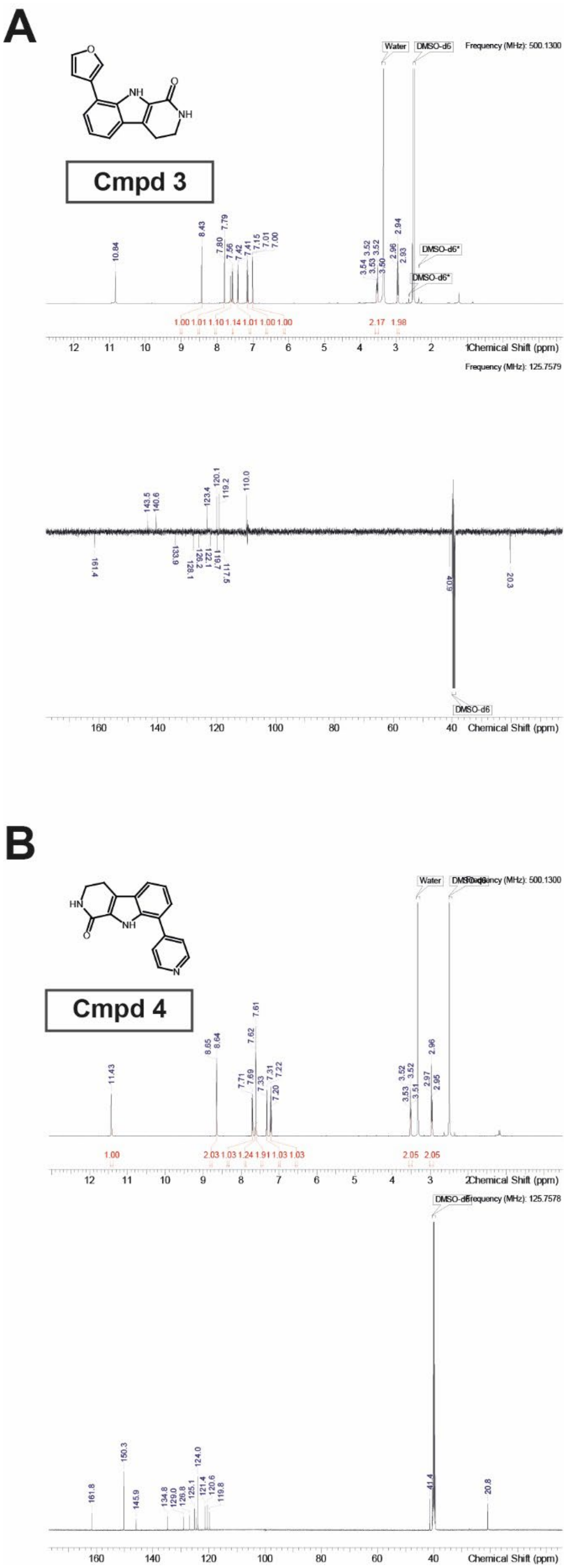


FIGURE S4. $^{1}H$ and $^{13}C$ NMR spectra of (A) Cmpd **3** and (B) Cmpd **4**.

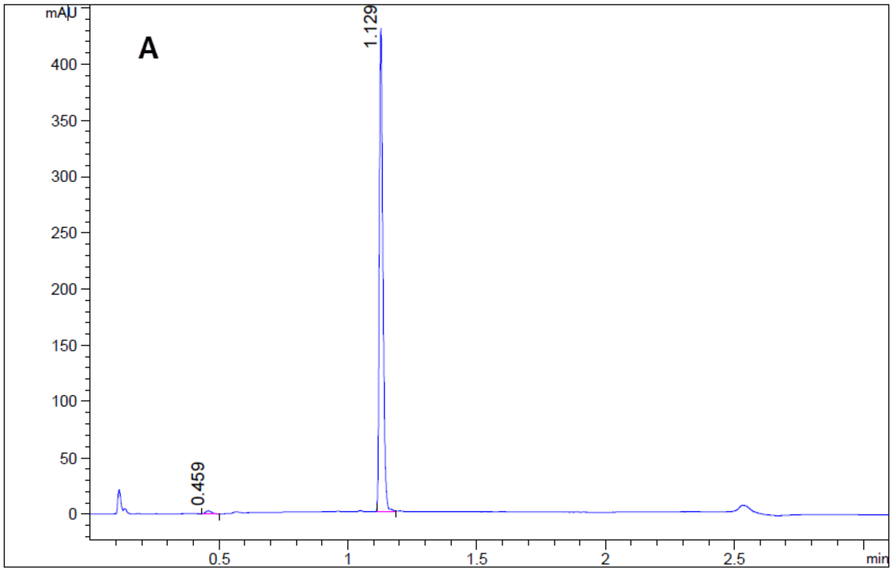


```
---------------------------------------------------------------------------
RT:   0.46 min                          Area %:     1.0 %
RT:   1.13 min                          Area %:    99.0 %
---------------------------------------------------------------------------
```

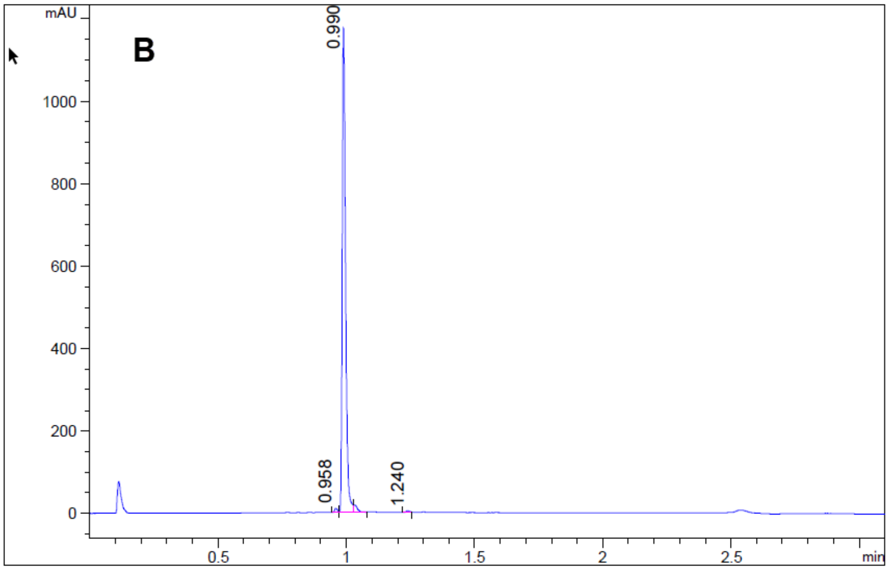


```
---------------------------------------------------------------------------
RT:   0.96 min                          Area %:     0.7 %
RT:   0.99 min                          Area %:    97.3 %
RT:   1.03 min                          Area %:     1.7 %
RT:   1.24 min                          Area %:     0.3 %
---------------------------------------------------------------------------
```

FIGURE S5. HPLC chromatogram of (A) Cmpd **3** and (B) Cmpd **4**.

Supplementary Table S1. Disclosed target list published by Boehringer Ingelheim listed with assay technologies used in this study and $IC_{50}$ values for measured MIIS. Number of targets per published target include screen and counterscreens. Colour Code for IC50: **IC50 < 1µM**, **IC50 between 1 and 10µM**, IC50 > 10µM.

| Published target | Assay technology | # of targets | MIIS | Target #1 $IC_{50}$ [µM] | Target #2 $IC_{50}$ [µM] | Target #3 $IC_{50}$ [µM] | Target #4 $IC_{50}$ [µM] |
|---|---|---|---|---|---|---|---|
| FKBP51 [1] | AlphaScreen | Target #1: FKBP51 | $PdCl_2$ | >10 | >10 | >10 | |
| | | Target #2: off-target | $AuCl_3$ | >10 | >10 | >10 | |
| | | Target #3: off target | $Ag(CH_3COO)$ | >10 | >10 | >10 | |
| | | | $FeCl_2$ | >10 | >10 | >10 | |
| | | | $FeCl_3$ | >10 | >10 | >10 | |
| | | | $SnCl_2$ | 1-10 | >10 | >10 | |
| | | | $CuCl_2$ | >10 | >10 | >10 | |
| | | | $NiCl_2$ | >10 | >10 | >10 | |
| | | | $Al(NO_3)_3$ | <1 | 1-10 | >10 | |
| | | | $MnCl_2$ | >10 | >10 | >10 | |
| | | | $ZnCl_2$ | >10 | >10 | >10 | |
| | | | $CoCl_2$ | >10 | >10 | >10 | |
| | | | $MgCl_2$ | >10 | >10 | >10 | |
| KRAS [2] | AlphaScreen | Target #1: KRAS GDP | $PdCl_2$ | <1 | <1 | >10 | |
| | | Target #2: KRAS GTP | $AuCl_3$ | 1-10 | 1-10 | >10 | |
| | | Target #3: off target | $Ag(CH_3COO)$ | >10 | >10 | >10 | |
| | | | $FeCl_2$ | 1-10 | 1-10 | >10 | |
| | | | $FeCl_3$ | 1-10 | 1-10 | >10 | |
| | | | $SnCl_2$ | >10 | 1-10 | >10 | |
| | | | $CuCl_2$ | >10 | >10 | >10 | |
| | | | $NiCl_2$ | >10 | >10 | >10 | |
| | | | $Al(NO_3)_3$ | >10 | >10 | >10 | |
| | | | $MnCl_2$ | >10 | >10 | >10 | |
| | | | $ZnCl_2$ | >10 | >10 | >10 | |
| | | | $CoCl_2$ | >10 | >10 | >10 | |
| | | | $MgCl_2$ | >10 | 1-10 | >10 | |
| MDM2 [3] | AlphaScreen | Target #1: MDM2 | $PdCl_2$ | >10 | >10 | >10 | |
| | | Target #2: off-target | $AuCl_3$ | >10 | >10 | >10 | |
| | | Target #3: off target | $Ag(CH_3COO)$ | >10 | >10 | >10 | |
| | | | $FeCl_2$ | >10 | | >10 | |
| | | | $FeCl_3$ | >10 | >10 | >10 | |
| | | | $SnCl_2$ | >10 | >10 | >10 | |
| | | | $CuCl_2$ | >10 | >10 | >10 | |
| | | | $NiCl_2$ | >10 | >10 | >10 | |
| | | | $Al(NO_3)_3$ | >10 | >10 | >10 | |
| | | | $MnCl_2$ | >10 | >10 | >10 | |
| | | | $ZnCl_2$ | >10 | >10 | >10 | |
| | | | $CoCl_2$ | >10 | >10 | >10 | |
| | | | $MgCl_2$ | >10 | >10 | >10 | |
| SOS1 [4] | AlphaScreen | Target #1: SOS1 | $PdCl_2$ | <1 | >10 | | |
| | | Target #2: off-target | $AuCl_3$ | <1 | >10 | | |
| | | | $Ag(CH_3COO)$ | <1 | >10 | | |
| | | | $FeCl_2$ | >10 | >10 | | |
| | | | $FeCl_3$ | >10 | >10 | | |
| | | | $SnCl_2$ | >10 | >10 | | |
| | | | $CuCl_2$ | 1-10 | >10 | | |
| | | | $NiCl_2$ | >10 | >10 | | |
| | | | $Al(NO_3)_3$ | >10 | >10 | | |
| | | | $MnCl_2$ | >10 | >10 | | |
| | | | $ZnCl_2$ | >10 | >10 | | |
| | | | $CoCl_2$ | >10 | >10 | | |
| | | | $MgCl_2$ | >10 | >10 | | |
| UPA-UPAR [5] | AlphaScreen | Target #1: UPAR | $PdCl_2$ | <1 | <1 | | |
| | | Target #2: off-target | $AuCl_3$ | 1-10 | 1-10 | | |
| | | | $FeCl_2$ | >10 | >10 | | |
| | | | $FeCl_3$ | >10 | >10 | | |
| | | | $SnCl_2$ | >10 | >10 | | |
| | | | $CuCl_2$ | <1 | >10 | | |
| | | | $NiCl_2$ | <1 | >10 | | |
| | | | $Al(NO_3)_3$ | >10 | >10 | | |
| | | | $MnCl_2$ | >10 | >10 | | |
| | | | $ZnCl_2$ | <1 | >10 | | |
| | | | $CoCl_2$ | 1-10 | >10 | | |
| | | | $MgCl_2$ | >10 | >10 | | |

| | | | | | | | |
|---|---|---|---|---|---|---|---|
| WRN [6] | ADP-Glo | Target #1: WRN (int.) | $PdCl_2$ | **<1** | **1-10** | **<1** | >10 |
| | | Target #2: WRN (ext.) | $AuCl_3$ | >10 | **1-10** | >10 | >10 |
| | | Target #3: off-target (int.) | $Ag(CH_3COO)$ | >10 | >10 | >10 | >10 |
| | | Target #4: off target (ext.) | $FeCl_2$ | >10 | >10 | >10 | >10 |
| | | | $FeCl_3$ | >10 | >10 | >10 | >10 |
| | | | $SnCl_2$ | >10 | >10 | >10 | >10 |
| | | | $CuCl_2$ | >10 | **1-10** | >10 | >10 |
| | | | $NiCl_2$ | >10 | >10 | >10 | >10 |
| | | | $Al(NO_3)_3$ | >10 | >10 | **1-10** | >10 |
| | | | $MnCl_2$ | >10 | >10 | >10 | >10 |
| | | | $ZnCl_2$ | **1-10** | >10 | >10 | >10 |
| | | | $CoCl_2$ | >10 | >10 | >10 | >10 |
| | | | $MgCl_2$ | >10 | >10 | >10 | >10 |
| BCL6 [7] | Fluorescence Polarization | Target #1: BCL6 | $PdCl_2$ | >10 | | | |
| | | | $AuCl_3$ | >10 | | | |
| | | | $Ag(CH_3COO)$ | >10 | | | |
| | | | $FeCl_2$ | >10 | | | |
| | | | $FeCl_3$ | >10 | | | |
| | | | $SnCl_2$ | >10 | | | |
| | | | $CuCl_2$ | >10 | | | |
| | | | $NiCl_2$ | >10 | | | |
| | | | $Al(NO_3)_3$ | >10 | | | |
| | | | $MnCl_2$ | >10 | | | |
| | | | $ZnCl_2$ | >10 | | | |
| | | | $CoCl_2$ | >10 | | | |
| | | | $MgCl_2$ | >10 | | | |
| beta-Catenin [8] | TR-FRET | Target #1: beta-Catenin | $PdCl_2$ | >10 | | | |
| | | | $AuCl_3$ | **<1** | | | |
| | | | $Ag(CH_3COO)$ | **<1** | | | |
| | | | $FeCl_2$ | >10 | | | |
| | | | $FeCl_3$ | >10 | | | |
| | | | $SnCl_2$ | >10 | | | |
| | | | $CuCl_2$ | >10 | | | |
| | | | $NiCl_2$ | >10 | | | |
| | | | $Al(NO_3)_3$ | >10 | | | |
| | | | $MnCl_2$ | >10 | | | |
| | | | $ZnCl_2$ | >10 | | | |
| | | | $CoCl_2$ | >10 | | | |
| | | | $MgCl_2$ | >10 | | | |
| SOS1 [4] | TR-FRET | Target #1: SOS1 | $PdCl_2$ | **1-10** | | | |
| | | | $AuCl_3$ | **<1** | | | |
| | | | $Ag(CH_3COO)$ | **<1** | | | |
| | | | $FeCl_2$ | **1-10** | | | |
| | | | $FeCl_3$ | **1-10** | | | |
| | | | $SnCl_2$ | **1-10** | | | |
| | | | $CuCl_2$ | >10 | | | |
| | | | $NiCl_2$ | >10 | | | |
| | | | $Al(NO_3)_3$ | >10 | | | |
| | | | $MnCl_2$ | >10 | | | |
| | | | $ZnCl_2$ | >10 | | | |
| | | | $CoCl_2$ | >10 | | | |
| | | | $MgCl_2$ | >10 | | | |
| KRAS [2] | TR-FRET | Target #1: KRAS | $PdCl_2$ | **1-10** | | | |
| | | | $AuCl_3$ | **<1** | | | |
| | | | $Ag(CH_3COO)$ | **<1** | | | |
| | | | $FeCl_2$ | >10 | | | |
| | | | $FeCl_3$ | **1-10** | | | |
| | | | $SnCl_2$ | **1-10** | | | |
| | | | $CuCl_2$ | >10 | | | |
| | | | $NiCl_2$ | >10 | | | |
| | | | $Al(NO_3)_3$ | >10 | | | |
| | | | $MnCl_2$ | >10 | | | |
| | | | $ZnCl_2$ | >10 | | | |
| | | | $CoCl_2$ | >10 | | | |
| | | | $MgCl_2$ | >10 | | | |
| LSD1 [9] | DELFIA | Target #1: LSD1 | $PdCl_2$ | **1-10** | | | |
| | | | $AuCl_3$ | **1-10** | | | |
| | | | $Ag(CH_3COO)$ | **1-10** | | | |
| | | | $FeCl_2$ | >10 | | | |
| | | | $FeCl_3$ | >10 | | | |
| | | | $SnCl_2$ | >10 | | | |
| | | | $CuCl_2$ | >10 | | | |

| | | | | | | | |
|---|---|---|---|---|---|---|---|
| | | | $NiCl_2$ | **1-10** | | | |
| | | | $Al(NO_3)_3$ | **1-10** | | | |
| | | | $MnCl_2$ | >10 | | | |
| | | | $ZnCl_2$ | >10 | | | |
| | | | $MgCl_2$ | >10 | | | |
| NSD3 [10] | TR-FRET | Target #1: NSD3 | $PdCl_2$ | >10 | | | |
| | | | $AuCl_3$ | **<1** | | | |
| | | | $Ag(CH_3COO)$ | **1-10** | | | |
| | | | $FeCl_2$ | >10 | | | |
| | | | $FeCl_3$ | >10 | | | |
| | | | $SnCl_2$ | **<1** | | | |
| | | | $CuCl_2$ | >10 | | | |
| | | | $NiCl_2$ | >10 | | | |
| | | | $Al(NO_3)_3$ | >10 | | | |
| | | | $MnCl_2$ | >10 | | | |
| | | | $ZnCl_2$ | >10 | | | |
| | | | $CoCl_2$ | >10 | | | |
| | | | $MgCl_2$ | >10 | | | |
| EGFR [11] | MALDI | Target #1: EGFR | $PdCl_2$ | >10 | | | |
| | | | $AuCl_3$ | >10 | | | |
| | | | $Ag(CH_3COO)$ | >10 | | | |
| | | | $FeCl_2$ | >10 | | | |
| | | | $FeCl_3$ | >10 | | | |
| | | | $SnCl_2$ | >10 | | | |
| | | | $CuCl_2$ | >10 | | | |
| | | | $NiCl_2$ | >10 | | | |
| | | | $Al(NO_3)_3$ | >10 | | | |
| | | | $MnCl_2$ | >10 | | | |
| | | | $ZnCl_2$ | >10 | | | |
| | | | $CoCl_2$ | >10 | | | |
| | | | $MgCl_2$ | >10 | | | |

Supplementary Table S2. Xray data collection and refinement statistics for Cmpd **3** and Cmpd **4**.

| | Cmpd **3** | Cmpd **4** |
|---|---|---|
| Data Collection | | |
| Space group | P 1 21 1 | P 1 21 1 |
| Cell dimensions | | |
| a, b, c (Å) | 42.72 72.34 54.51 | 42.67 71.91 54.53 |
| α, β, γ (°) | 90.00, 103.45, 90.00 | 90.00, 103.41, 90.00 |
| Resolution (Å) | 53.01–1.16 (1.28–1.16)* | 53.04–1.31 (1.39–1.31)* |
| Unique reflections | 71438 (338)* | 64754 (1343)* |
| CC 1/2 | 0.99 (0.69)* | 0.99 (0.56)* |
| Rmerge | 0.03 (0.64)* | 0.04 (0.80) * |
| I/σI | 15.40 (1.70)* | 11.60 (1.50)* |
| Completeness (%) | 93.10 (70.80)* | 93.20 (51.90)* |
| Redundancy | 3.40 (3.50)* | 3.40 (3.60)* |
| Refinement | | |
| Resolution (Å) | 1.16 | 1.31 |
| Rwork/Rfree | 17.86 / 20.22 | 16.48 / 19.15 |
| Average B factor ($Å^2$) | 21.64 | 23.00 |
| Number of non-hydrogen atoms | 3067 | 3142 |
| macromolecules | 2744 | 2740 |
| ligands | 161 | 132 |
| solvent | 219 | 316 |
| Rotamer outliers [%] | 0.97 | 0.65 |
| Ramachandran favored [%] | 96.07 | 96.67 |
| Ramachandran allowed [%] | 3.93 | 3.33 |
| Ramachandran outliers [%] | 0.00 | 0.00 |
| Root Mean Square Deviations | | |
| Bond lengths (Å) | 0.01 | 0.01 |
| Bond angles (°) | 1.04 | 1.05 |
| Clashscore | 2.31 | 2.15 |

* Values in parentheses represent the highest-resolution shell.

References

(1) Kästle, M.; Kistler, B.; Lamla, T.; Bretschneider, T.; Lamb, D.; Nicklin, P.; Wyatt, D. FKBP51 Modulates Steroid Sensitivity and NFκB Signalling: A Novel Anti-inflammatory Drug Target. *Eur. J. Immunol.* **2018**, *48* (11), 1904–1914. https://doi.org/10.1002/eji.201847699.

(2) Kessler, D.; Gmachl, M.; Mantoulidis, A.; Martin, L. J.; Zoephel, A.; Mayer, M.; Gollner, A.; Covini, D.; Fischer, S.; Gerstberger, T.; Gmaschitz, T.; Goodwin, C.; Greb, P.; Häring, D.; Hela, W.; Hoffmann, J.; Karolyi-Oezguer, J.; Knesl, P.; Kornigg, S.; Koegl, M.; Kousek, R.; Lamarre, L.; Moser, F.; Munico-Martinez, S.; Peinsipp, C.; Phan, J.; Rinnenthal, J.; Sai, J.; Salamon, C.; Scherbantin, Y.; Schipany, K.; Schnitzer, R.; Schrenk, A.; Sharps, B.; Siszler, G.; Sun, Q.; Waterson, A.; Wolkerstorfer, B.; Zeeb, M.; Pearson, M.; Fesik, S. W.; McConnell, D. B. Drugging an Undruggable Pocket on KRAS. *P Natl Acad Sci Usa* **2019**, *116* (32), 15823–15829. https://doi.org/10.1073/pnas.1904529116.

(3) Gollner, A.; Rudolph, D.; Arnhof, H.; Bauer, M.; Blake, S. M.; Boehmelt, G.; Cockroft, X.-L.; Dahmann, G.; Ettmayer, P.; Gerstberger, T.; Karolyi-Oezguer, J.; Kessler, D.; Kofink, C.; Ramharter, J.; Rinnenthal, J.; Savchenko, A.; Schnitzer, R.; Weinstabl, H.; Weyer-Czernilofsky, U.; Wunberg, T.; McConnell, D. B. Discovery of Novel Spiro[3H-indole-3,2′-Pyrrolidin]-2(1H)-one Compounds as Chemically Stable and Orally Active Inhibitors of the MDM2–P53 Interaction. *J. Med. Chem.* **2016**, *59* (22), 10147–10162. https://doi.org/10.1021/acs.jmedchem.6b00900.

(4) Ramharter, J.; Kessler, D.; Ettmayer, P.; Hofmann, M. H.; Gerstberger, T.; Gmachl, M.; Wunberg, T.; Kofink, C.; Sanderson, M.; Arnhof, H.; Bader, G.; Rumpel, K.; Zöphel, A.; Schnitzer, R.; Böttcher, J.; O'Connell, J. C.; Mendes, R. L.; Richard, D.; Pototschnig, N.; Weiner, I.; Hela, W.; Hauer, K.; Haering, D.; Lamarre, L.; Wolkerstorfer, B.; Salamon, C.; Werni, P.; Munico-Martinez, S.; Meyer, R.; Kennedy, M. D.; Kraut, N.; McConnell, D. B. One Atom Makes All the Difference: Getting a Foot in the Door between SOS1 and KRAS. *J. Med. Chem.* **2021**, *64* (10), 6569–6580. https://doi.org/10.1021/acs.jmedchem.0c01949.

(5) Cheng, Y.; Hall, T. R.; Xu, X.; Yung, I.; Souza, D.; Zheng, J.; Schiele, F.; Hoffmann, M.; Mbow, M. L.; Garnett, J. P.; Li, J. Targeting UPA-UPAR Interaction to Improve Intestinal Epithelial Barrier Integrity in Inflammatory Bowel Disease. *Ebiomedicine* **2022**, *75*, 103758. https://doi.org/10.1016/j.ebiom.2021.103758.

(6) Lieb, S.; Blaha-Ostermann, S.; Kamper, E.; Rippka, J.; Schwarz, C.; Ehrenhöfer-Wölfer, K.; Schlattl, A.; Wernitznig, A.; Lipp, J. J.; Nagasaka, K.; Lelij, P. van der; Bader, G.; Koi, M.; Goel, A.; Neumüller, R. A.; Peters, J.-M.; Kraut, N.; Pearson, M. A.; Petronczki, M.; Wöhrle, S. Werner Syndrome Helicase Is a Selective Vulnerability of Microsatellite Instability-High Tumor Cells. *Elife* **2019**, *8*, e43333. https://doi.org/10.7554/elife.43333.

(7) Kerres, N.; Steurer, S.; Schlager, S.; Bader, G.; Berger, H.; Caligiuri, M.; Dank, C.; Engen, J. R.; Ettmayer, P.; Fischerauer, B.; Flotzinger, G.; Gerlach, D.; Gerstberger, T.; Gmaschitz, T.; Greb, P.; Han, B.; Heyes, E.; Iacob, R. E.; Kessler, D.; Kölle, H.; Lamarre, L.; Lancia, D. R.; Lucas, S.; Mayer, M.; Mayr, K.; Mischerikow, N.; Mück, K.; Peinsipp, C.; Petermann, O.; Reiser, U.; Rudolph, D.; Rumpel, K.; Salomon, C.; Scharn, D.; Schnitzer, R.; Schrenk, A.; Schweifer, N.; Thompson, D.; Traxler, E.; Varecka, R.; Voss, T.; Weiss-Puxbaum, A.; Winkler, S.; Zheng, X.; Zoephel, A.; Kraut, N.; McConnell, D.; Pearson, M.; Koegl, M. Chemically Induced Degradation of the Oncogenic Transcription Factor BCL6. *Cell Rep.* **2017**, *20* (12), 2860–2875. https://doi.org/10.1016/j.celrep.2017.08.081.

(8) Kessler, D.; Mayer, M.; Zahn, S. K.; Zeeb, M.; Wöhrle, S.; Bergner, A.; Bruchhaus, J.; Ciftci, T.; Dahmann, G.; Dettling, M.; Döbel, S.; Fuchs, J. E.; Geist, L.; Hela, W.; Kofink, C.; Kousek, R.; Moser, F.; Puchner, T.; Rumpel, K.; Scharnweber, M.; Werni, P.; Wolkerstorfer, B.; Breitsprecher, D.; Baaske, P.; Pearson, M.; McConnell, D. B.; Böttcher, J. Getting a Grip on the Undrugged: Targeting Β-Catenin with Fragment-Based Methods. *Chemmedchem* **2021**, *16* (9), 1420–1424. https://doi.org/10.1002/cmdc.202000839.

(9) Castex, J.; Willmann, D.; Kanouni, T.; Arrigoni, L.; Li, Y.; Friedrich, M.; Schleicher, M.; Wöhrle, S.; Pearson, M.; Kraut, N.; Méret, M.; Manke, T.; Metzger, E.; Schüle, R.; Günther, T. Inactivation of Lsd1 Triggers Senescence in Trophoblast Stem Cells by Induction of Sirt4. *Cell Death Dis* **2017**, *8* (2), e2631–e2631. https://doi.org/10.1038/cddis.2017.48.

(10) Böttcher, J.; Dilworth, D.; Reiser, U.; Neumüller, R. A.; Schleicher, M.; Petronczki, M.; Zeeb, M.; Mischerikow, N.; Allali-Hassani, A.; Szewczyk, M. M.; Li, F.; Kennedy, S.; Vedadi, M.; Barsyte-Lovejoy, D.; Brown, P. J.; Huber, K. V. M.; Rogers, C. M.; Wells, C. I.; Fedorov, O.; Rumpel, K.; Zoephel, A.; Mayer, M.; Wunberg, T.; Böse, D.; Zahn, S.; Arnhof, H.; Berger, H.; Reiser, C.; Hörmann, A.; Krammer, T.; Corcokovic, M.; Sharps, B.; Winkler, S.; Häring, D.; Cockcroft, X.-L.; Fuchs, J. E.; Müllauer, B.; Weiss-Puxbaum, A.; Gerstberger, T.; Boehmelt, G.; Vakoc, C. R.; Arrowsmith, C. H.; Pearson, M.; McConnell, D. B. Fragment-Based Discovery of a Chemical Probe for the PWWP1 Domain of NSD3. *Nat. Chem. Biol.* **2018**, *15* (8), 822–829. https://doi.org/10.1038/s41589-019-0310-x.

(11) Engelhardt, H.; Böse, D.; Petronczki, M.; Scharn, D.; Bader, G.; Baum, A.; Bergner, A.; Chong, E.; Döbel, S.; Egger, G.; Engelhardt, C.; Ettmayer, P.; Fuchs, J. E.; Gerstberger, T.; Gonnella, N.; Grimm, A.; Grondal, E.; Haddad, N.; Hopfgartner, B.; Kousek, R.; Krawiec, M.; Kriz, M.; Lamarre, L.; Leung, J.; Mayer, M.; Patel, N. D.; Simov, B. P.; Reeves, J. T.; Schnitzer, R.; Schrenk, A.; Sharps, B.; Solca, F.; Stadtmüller, H.; Tan, Z.; Wunberg, T.; Zoephel, A.; McConnell, D. B. Start Selective and Rigidify: The Discovery Path toward a Next Generation of EGFR Tyrosine Kinase Inhibitors. *J. Med. Chem.* **2019**, *62* (22), 10272–10293. https://doi.org/10.1021/acs.jmedchem.9b01169.
